# Monocytes/macrophages support mammary tumor invasivity by co-secreting lineage-specific EGFR ligands and a STAT3 activator

**DOI:** 10.1186/1471-2407-13-197

**Published:** 2013-04-18

**Authors:** Philip Vlaicu, Philipp Mertins, Thomas Mayr, Peter Widschwendter, Beyhan Ataseven, Bernhard Högel, Wolfgang Eiermann, Pjotr Knyazev, Axel Ullrich

**Affiliations:** 1 Department of Molecular Biology, Max Planck Institute of Biochemistry, Martinsried 82152, Germany; 2 Department of Gynecology and Obstetrics, Red Cross Hospital, Munich 80637, Germany; 3 Current address: Proteomics Platform, Broad Institute of MIT and Harvard, Cambridge, MA 02142, USA; 4 Current address: University Hospital for Women, University of Tuebingen, Tuebingen 72076, Germany

**Keywords:** TAM, Macrophage, Monocyte, Breast, Carcinoma, Patient, EGFR, STAT3, Marker, Progression

## Abstract

**Background:**

Tumor-associated macrophages (TAM) promote malignant progression, yet the repertoire of oncogenic factors secreted by TAM has not been clearly defined. We sought to analyze which EGFR- and STAT3-activating factors are secreted by monocytes/macrophages exposed to tumor cell-secreted factors.

**Methods:**

Following exposure of primary human monocytes and macrophages to supernatants of a variety of tumor cell lines, we have analyzed transcript and secreted protein levels of EGFR family ligands and of STAT3 activators. To validate our findings, we have analyzed TAM infiltration levels, systemic and local protein levels as well as clinical data of primary breast cancer patients.

**Results:**

Primary human monocytes and macrophages respond to tumor cell-derived factors by secreting EGFR- and STAT3-activating ligands, thus inducing two important oncogenic pathways in carcinoma cells. Tumor cell-secreted factors trigger two stereotype secretory profiles in peripheral blood monocytes and differentiated macrophages: monocytes secrete epiregulin (EREG) and oncostatin-M (OSM), while macrophages secrete heparin-binding EGF-like growth factor (HB-EGF) and OSM. HB-EGF and OSM cooperatively induce tumor cell chemotaxis. HB-EGF and OSM are co-expressed by TAM in breast carcinoma patients, and plasma levels of both ligands correlate strongly. Elevated HB-EGF levels accompany TAM infiltration, tumor growth and dissemination in patients with invasive disease.

**Conclusions:**

Our work identifies systemic markers for TAM involvement in cancer progression, with the potential to be developed into molecular targets in cancer therapy.

## Background

Molecular targeting of cancer cells has proven to be an extremely effective approach in tumor therapy. However, the inherent genomic instability of tumor cells promotes the establishment of therapy-refractory cancer cell clones during the course of treatment [1].

In addition, recent studies of the tumor microenvironment have emphasised that cancer cells effectively reprogram infiltrating immune cells, to the point where they support malignant progression [2]. Tumor-associated macrophages (TAM) are key participants in the crosstalk between tumor and immune cells. Thus, these genomically stable cells represent promising targets for anticancer therapy. TAM density correlates with poor prognosis in a considerable number of human cancers [3]. Their best studied contribution to malignancy is the involvement in tumor progression. In a mouse breast carcinoma model, Pollard and colleagues have shown that macrophage (MΦ) ablation inhibits metastasis [4]. MΦ exert important functions as trophic agents by secreting a plethora of growth factors and metalloproteases [5,6]. With TAM being the most thoroughly studied components of the innate immune system, less is known about the impact of tumor-associated monocytes on tumor progression. Yet, populations of Tie2^+^ infiltrating monocytes promote angiogenesis in a mouse tumor model [7].

We studied the influence of tumor cells on ligand transcription and secretion patterns of monocytes and differentiated macrophages. Given the pivotal roles the oncogenic STAT3 and EGFR pathways play in tumor development [8,9], we focused primarily on the repertoire of interleukin-6-like STAT3 activators and EGFR agonists secreted by tumor-primed monocytes and MΦ. Apart its founder protein, the interleukin-6 cytokine family includes interleukin-11 (IL-11), leukemia inhibitory factor (LIF), ciliary neurotrophic factor (CNTF), cardiotrophin-1 (CT-1) and oncostatin-M (OSM). The group of epidermal growth factor-like ligands contains seven EGFR agonists: amphiregulin (AREG), betacellulin (BTC), epidermal growth factor (EGF), epigen (EPGN), epiregulin (EREG), heparin-binding EGF-like growth factor (HB-EGF) and transforming growth factor alpha (TGFα). Several lines of evidence point to a role of TAM-secreted EGFR ligands in malignant progression. McGee and colleagues provided clues for EGF production by TAM in breast carcinoma biopsies [10]. TAM number and EGFR expression levels correlate in human breast tumors [11], and TAM induce the EGFR-dependent migration of tumor cells in a murine breast carcinoma model [12]. However, the exact repertoire of TAM-secreted EGFR agonists is unknown, as are the related regulatory pathways.

Our work demonstrates that in response to tumor cell-derived factors, mononuclear cells in turn secrete oncogenic ligands. Subsequent to priming with tumor cell-secreted factors, human primary MΦ and peripheral blood monocytes (PBMC) both secrete OSM, but each cell type in combination with one specific EGFR agonist. These findings reveal characteristic secretory patterns of tumor-primed MΦ and PBMC in vitro, regardless of tumor etiology.

## Methods

### Ethics statement

This study was approved by the responsible ethics committee of the Ludwig-Maximilians-University, Munich, project nr. 189-11.

### Cell lines

HL-60, MDA-MB-231, T-47D, MCF7, MDA-MB-468, MDA-MB-435S, SCC-9 and MCF 10A cell lines were obtained from the American Type Culture Collection (Manassas, VA). Primary HMEC were obtained from Clonetics (Basel, Switzerland) and cultured in MEGM® medium (Lonza, Walkersville, MD). HL-60, MDA-MB-231, MCF7, MDA-MB-468 and MDA-MB-435S cells were cultured in RPMI1640 containing 10% heat-inactivated fetal bovine serum (Invitrogen, Grand Island, NY). T-47D cells were cultured in RPMI1640 containing 10% heat-inactivated fetal bovine serum and 10 μg/ml bovine insulin (Sigma, St. Louis, MO). SCC-9 cells were cultured in DMEM/Ham’s F-12 (1:1) containing 10% heat-inactivated fetal bovine serum and 0.4 μg/ml hydrocortisone (Sigma). MCF 10A cells were cultured in DMEM/Ham’s F-12 (1:1) containing 5% heat-inactivated horse serum (Invitrogen), 0.5 μg/ml hydrocortisone, 20 ng/ml EGF (PeproTech, Hamburg, Germany), 10 μg/ml bovine insulin, 0.1 μg/ml cholera toxin (Sigma). All culture media contained 100 Units/ml Penicillin and 0.1 mg/ml Streptomycin (PAA, Pasching, Austria). Cells were cultured at 37°C in humidified air under 5% CO_2_ and were confirmed to be devoid of mycoplasma.

### Monocyte isolation and macrophage differentiation

Peripheral blood was collected from healthy human donors after obtaining informed written consent. 15 ml of EDTA-treated blood were layered over a Ficoll-Paque™ PLUS column (GE Healthcare, Piscataway, NJ) and centrifuged at 400 g for 30 minutes, at 18°C. Leukocytes were washed twice with ice-cold PBS and transferred into RPMI containing 10% heat-inactivated fetal bovine serum and 5% active human serum. Following adherence to gelatine-coated culture plates (Sigma) that had been freshly precoated with human plasma, monocytes were washed 5 times with warm washing buffer (RPMI and PBS; 1:1), then detached using 5 mM EDTA in washing buffer. Cells were collected, centrifuged at 400 g for 15 minutes at 4°C then washed twice with ice-cold washing buffer. PBMC were plated at a density of 1.0 × 10^6^ cells/ml in 6 well culture plates (Corning, Corning, NY) in monocyte culture medium (RPMI supplemented with 5% normal human male AB serum, Sigma). After 4 days of cultivation, cells were washed 3 times with RPMI and received fresh culture medium. Between day 4 and 10, medium was changed every third day. After 7 days of culture, macrophages had completed spontaneous differentiation.

### Migration assays

Unless otherwise stated, cells were plated into transwell migration inserts (BD Biosciences, Bedford, MA) using RPMI + 0.1% BSA. The lower compartments contained either serum-free MΦ supernatants, culture media or RPMI + 0.1% BSA with or without OSM/HB-EGF. Where indicated, a 1 hr preincubation with neutralising antibodies, DMSO or AG1478 was performed prior to cell plating. At the end of the migration period, cells were fixed and stained with 20% methanol, 0.5% crystal violet and cotton swabs were used to remove the cells from the upper side of the membrane. An Axio Observer.A1 microscope (Carl Zeiss, Jena, Germany) was used to record 5 micrographs per well. Photoshop CS4 (Adobe, San Jose, CA) and a proprietary script were used for automated quantification of migration 5 × 10^4^ SCC-9 cells were allowed to migrate for 6 hrs (chemotaxis) or 17 hrs (intrinsic motility). 1.5 × 10^5^ MCF 10A cells were allowed to migrate for 17 hrs. 1.5 × 10^5^ MCF7 cells were allowed to migrate for 48 hrs.

### Statistical analysis

Two-tailed Mann–Whitney U-Tests were used for qPCR and ELISA experiments. For immunoblot, motility and chemotaxis experiments we used the two-tailed Student’s T-Test. Differences were considered significant when p < 0.05. Data are presented as means ± SEM or SD.

## Results

### Tumor cells and primary mononuclear cells interact via secreted factors

**Figure 1:**
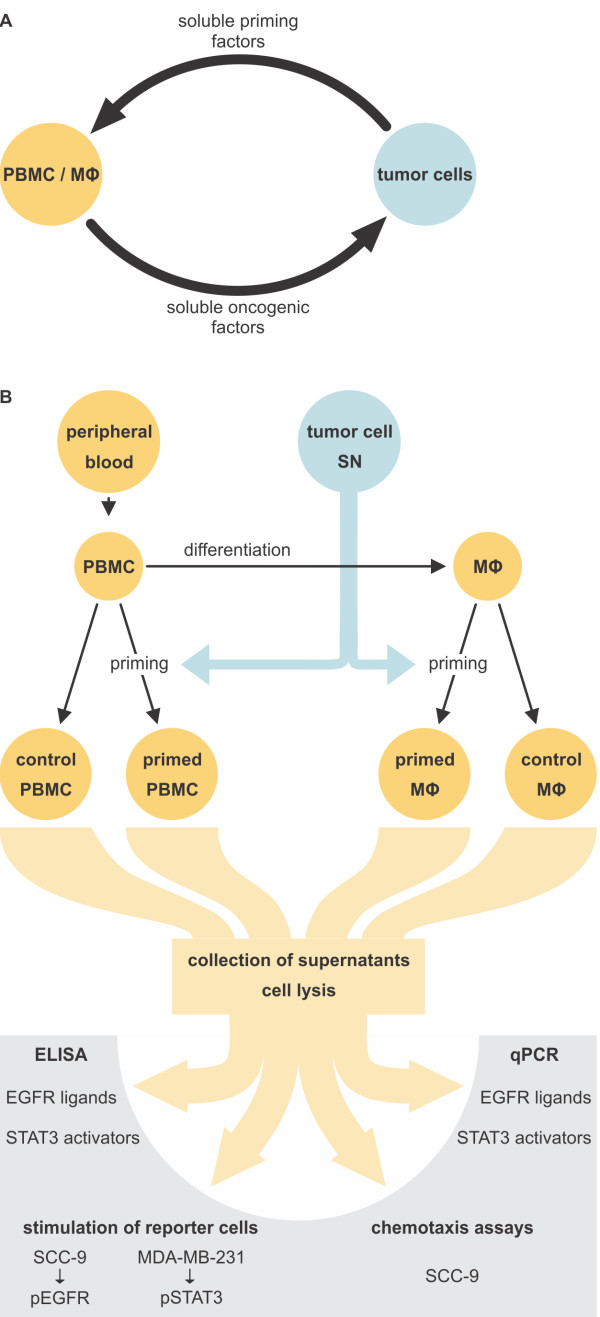
**Tumor cells and primary mononuclear cells interact via secreted factors.** (**A**). Model for the interaction between tumor cells and mononuclear cells. (**B**). Experimental approach for the study of effects of tumor cell supernatants (SN) on primary human PBMC and in vitro differentiated MΦ.

To analyse paracrine interactions via secreted factors, we designed an experimental setup based on the priming of primary human mononuclear cells with supernatants from a panel of human tumor and nontransformed cells. In contrast to direct coculture, such priming experiments permit a thorough analysis of factors secreted by both cell types, at protein and transcript level. Figure 1A provides our model of tumor cell/mononuclear cell interaction, based on mutually secreted factors. *In vivo*, tumor-derived soluble factors trigger the extravasation of PBMC in the vicinity of tumor cells [13]. This implies that tumor-primed PBMC already participate in the crosstalk between tumor cells and microenvironment. Following differentiation from extravasated PBMC, TAM are exposed to tumor-derived priming factors and release soluble oncogenic factors. In vitro, PBMC differentiate spontaneously to MΦ in the presence of human serum (Additional file 1: Figure S1A/B). To study the effects of tumor-secreted factors on primary human mononuclear cells in vitro, we primed PBMC or MΦ with tumor cell-supernatants for 24 hrs. Subsequent to priming, PBMC- or MΦ-conditioned media were used to stimulate reporter cells. MDA-MB-231 cells served as indicators of STAT3 activators present in the supernatants. SCC-9 cells, an established model for EGFR activation studies [14], were used as indicators of EGFR agonists secreted by PBMC/MΦ. Our experimental approach is presented in Figure 1B. Transcript analysis of PBMC and MΦ revealed that four EGFR agonists were expressed. In detail, we identified *AREG*, *EREG*, *HBEGF* and *TGFA* transcripts in both tested cell types. In contrast, *BTC*, *EGF* and *EPGN* transcripts were not detected. In addition, PBMC and MΦ expressed oncostatin-M, a STAT3 activator of the IL-6 family previously identified as a monocyte-secreted factor via mass spectrometric analysis (Additional file 1: Figure S1C and data not shown). While OSM was only expressed by myeloid cells, both OSM-specific receptors, OSMRβ and LIFR, were exclusively expressed by cells of epithelial origin (Additional file 1: Figure S2). This finding points at possible paracrine interactions between EGFR ligand- and OSM-expressing mononuclear cells and EGFR- and OSMRβ/LIFR-expressing carcinoma cells.

### Tumor cell-derived factors trigger divergent patterns of transcriptional activity and ligand secretion in PBMC and MΦ

**Figure 2:**
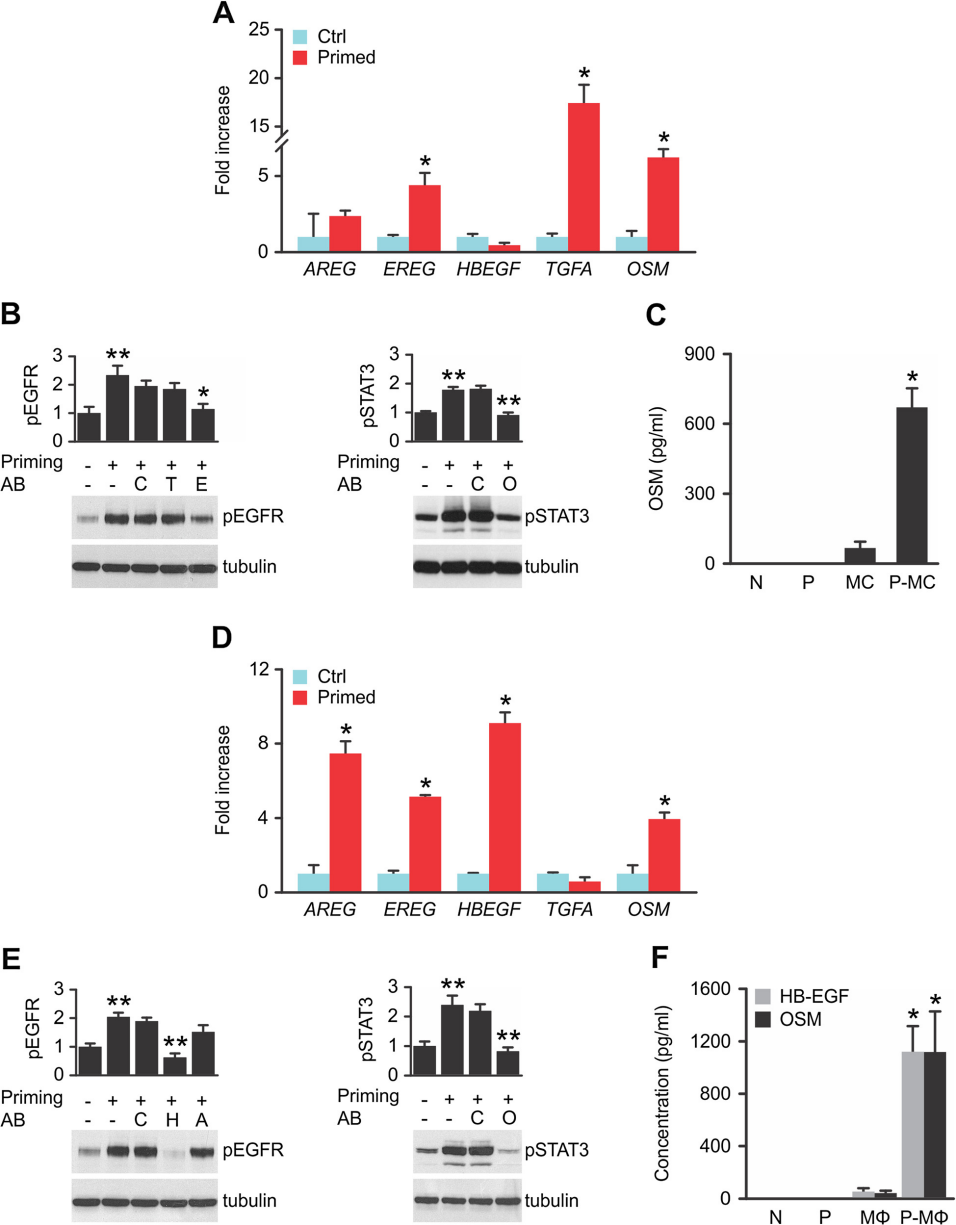
**PBMC and MΦ display divergent patterns of transcript induction and ligand secretion upon priming by tumor cells.** (**A**). Primed PBMC induce EREG, TGFA and OSM transcripts. qPCR of control-treated (Ctrl) or MDA-MB-231-primed (Primed) PBMC. Means ± 95% confidence intervals; *p < 0.05 by Mann-Whitney’s U-Test; n = 4. (**B**). Primed PBMC secrete EREG and OSM. PBMC supernatants were preincubated with control sera or blocking antibodies, then used to stimulate reporter cells in which pEGFR, pSTAT3 and tubulin levels were visualised. Western blots with densitometric analysis. Means ± SEM; n = 4; C: control Ig; T: TGFα blocking antibody; E: EREG blocking antibody; O: OSM blocking antibody; *p < 0.02; **p < 0.006 by Student’s T-Test. (**C**). Primed PBMC secrete elevated OSM levels. ELISA of control-treated or MDA-MB-231-primed PBMC. N: culture medium; P: priming medium; MC: control PBMC supernatant ; P-MC: primed PBMC supernatant; means ± SEM; n = 7; *p < 0.002 by Mann-Whitney’s U-Test. (**D**). Primed MΦ upregulate AREG, EREG, HBEGF and OSM transcription. qPCR of control-treated or MDA-MB-231-primed MΦ. Means ± 95% confidence intervals; *p < 0.02 by Mann-Whitney’s U-Test; n = 4. (**E**). Primed MΦ secrete HB-EGF and OSM. MΦ were control-treated or MDA-MB-231-primed for 24 hrs. MΦ supernatants were preincubated with control sera or blocking antibodies, then used to stimulate reporter cells in which pEGFR, pSTAT3 and tubulin levels were visualised. Western blots with densitometric analysis. Means ± SEM; pEGFR: n = 6; pSTAT3: n = 12; H: HB-EGF blocking antibody; A: AREG blocking antibody; O: OSM blocking antibody. **p < 0.00006 by Student’s T-Test. (**F**). Primed MΦ secrete elevated HB-EGF and OSM levels. ELISA of control-treated or MDA-MB-231-primed MΦ. N: culture medium; P: priming medium; MΦ: control macrophage supernatant; P-MΦ: primed macrophage supernatant; means ± SEM; n = 6; *p < 0.004 by Mann-Whitney’s U-Test.

To study the priming effects of tumor cell-secreted factors on primary human mononuclear cells, we used diluted MDA-MB-231 supernatants (1:2). Subsequent to priming, transcript and secreted protein levels of EGFR ligands and OSM were monitored. Priming by tumor cells upregulated *EREG*, *TGFA* and *OSM* transcripts in PBMC within 24 hrs, as shown by qPCR analysis (Figure 2A). Primed PBMC secreted factors that significantly induced pEGFR levels in the reporter cell line. We tested neutralising antibodies directed against AREG, EREG, HB-EGF and TGFα in order to identify the EGFR agonists secreted by primed PBMC. The used antibodies display no significant crossreactivity towards other EGFR ligands (Additional file 1: Figure S3). Epiregulin neutralisation by an inhibitory antibody reduced pEGFR to basal levels. Though highly expressed at transcript level, a TGFα blocking antibody had no effect (Figure 2B), and TGFα secretion was not detected in ELISA (data not shown). In contrast to TGFα, EREG plays a key role in EGFR activation in vitro. Highly elevated levels of OSM were detected in PBMC supernatants only after priming, while the protein was neither present in culture medium nor in tumor cell-conditioned medium used to prime PBMC (Figure 2C). Supernatants of primed PBMC significantly upregulated pSTAT3 levels in the reporter cell line, and STAT3 activation was reduced to basal levels by OSM blockage (Figure 2B). Priming of PBMC, enriched via adherence or purified via CD64/CD14 expression, resulted in similar levels of EGFR/STAT3 stimulation through EREG and OSM, confirming that these two factors were indeed produced and secreted by monocytic populations (Additional file 1: Figure S4A/B). Accordingly, pro-EREG was present in PBMC, but not in lymphocytes (Additional file 1: Figure S4C).

Together, these data indicate that that *EREG*, *TGFA* and *OSM* transcription is induced in PBMC upon priming with MDA-MB-231 supernatants. However, PBMC only secrete functionally active EREG and OSM proteins.

Priming of differentiated macrophages significantly increased *HBEGF*, *AREG* and *OSM* transcript levels (Figure 2D). *EREG* levels, though upregulated in primed MΦ, generally remain low in this cell type (Additional file 1: Figure S1C). Upon priming, MΦ supernatants significantly upregulated pEGFR levels in the reporter cell line, an effect which was completely blocked by an inhibitory antibody against HB-EGF. Though *AREG* transcription was highly upregulated, a neutralising antibody directed against AREG had no effect (Figure 2E). Supernatants of primed MΦ significantly elevated pSTAT3 levels in the reporter cell line, and an antibody blocking OSM function neutralised this effect (Figure 2E). Priming of differentiated MΦ for seven days provoked secretion of EGF family ligands and OSM to a similar extent (data not shown). Quantitation of secreted HB-EGF and OSM levels confirmed that secretion is significantly increased by priming (Figure 2F). In contrast, AREG was not secreted by primed MΦ (data not shown).

Taken together, these data indicate that, even though various EGFR ligands are transcriptionally induced, primed macrophages only secrete functionally active HB-EGF and OSM.

### Tumor-primed PBMC and MΦ display specific secretory patterns

Tumor-primed PBMC and MΦ secrete one specific EGFR agonist, respectively, in combination with OSM, the relevant STAT3 activator. We next asked whether priming with an expanded panel of tumor cell supernatants provokes similar response patterns in PBMC and MΦ. The panel contained MDA-MB-231 (231), T-47D (T47), MCF7 (7), MDA-MB-468 (468) invasive mammary and SCC-9 (SCC) squamous carcinoma cell lines. MCF 10A (10A) are derived from a benign breast lesion and primary HMEC (HM) represent healthy breast epithelium.

After priming with 231, T47, 468 and 10A, PBMC stereotypically co-secrete EGFR and STAT3 activators (Additional file 1: Figure S5A). OSM was released in significant quantities by tumor-primed PBMC (Additional file 1: Figure S5B). Experiments with neutralising antibodies confirmed that EREG and OSM were the only functional EGFR and STAT3 activators secreted by tumor-primed PBMC (Additional file 1: Figure S5C/D). An overview of the factors secreted by PBMC upon priming by various tumor cell lines is given in Table 1.

**Table 1:** Tumor-secreted factors induce specific secretory patterns in PBMC and MΦ

**Priming supernatant**	**PBMC**		**MΦ**	
	**EREG**	**OSM**	**HB-EGF**	**OSM**
MDA-MB-231	+	+	+	+
T-47D	+	+	+	+
MCF7	-	-	+	+
MDA-MB-468	+	+	-	-
SCC-9	-	-	+	+
HMEC	-	-	-	-
MCF 10A	+	+	-	-

PBMC and MΦ were primed with the indicated supernatants and secretion of EREG, HB-EGF and OSM was assessed. Detailed data are presented in Additional file 1: Figures S5, S6.

MΦ primed by 231, T47, 7 and SCC co-secreted considerable amounts of HB-EGF and OSM (Table 1, Additional file 1: Figure S6B), which were shown to be the only EGFR and STAT3 activators released by primed MΦ (Additional file 1: Figure S6C/D).

We observed clearly distinct priming capacities of tumor cell lines on primary mononuclear cells: primary breast epithelial cells (HM) derived from healthy donors neither convey a secretory response in PBMC nor in MΦ (Table 1, Additional file 1: Figure S5/S6). Selective PBMC priming was achieved by 10A and 468 cells, while 7 and SCC cells exclusively primed MΦ. 231 and T47 breast carcinoma cells primed PBMC as well as MΦ (Table 1). Thus, a number of tumor cell lines triggers PBMC and MΦ to co-secrete an EGFR agonist and a STAT3 activator. The secretory patterns of tumor-primed mononuclear cells are lineage-specific: PBMC co-secrete EREG and OSM, while MΦ co-secrete HB-EGF and OSM.

### HB-EGF and OSM are promigratory in epithelial cells

**Figure 3:**
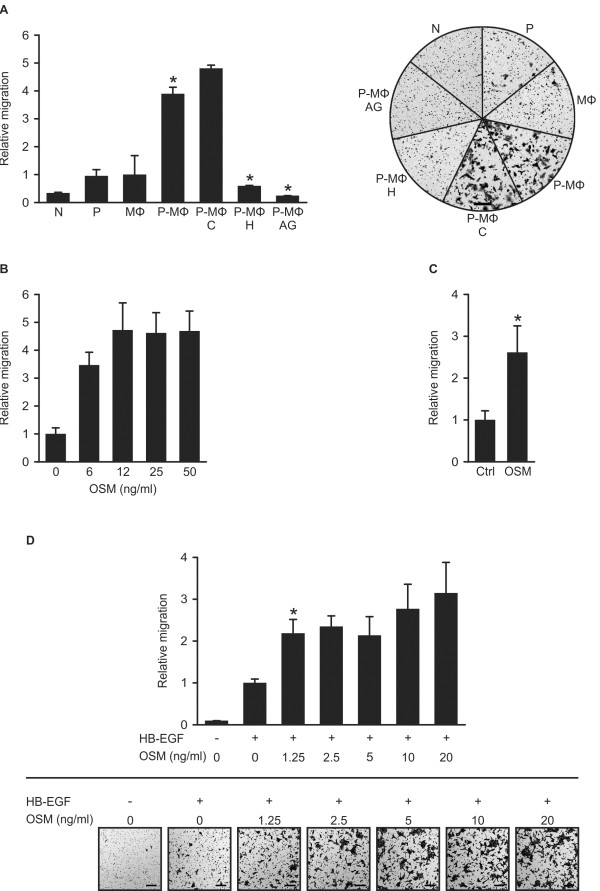
**HB-EGF and OSM promote epithelial cell migration.** (**A**). HB-EGF secreted by primed MΦ induces chemotaxis of SCC-9 cells. MΦ were cultured for 6 days in either control medium or 50% MDA-MB-231-conditioned medium. Subsequently, cells received 50% MDA-MB-231-conditioned serum-free medium or RPMI for 24 hrs. MΦ supernatants were collected and used as chemoattractants for SCC-9 cells in Boyden chamber assays. Where indicated, media were preincubated with control Ig (**C**), an HB-EGF blocking antibody (H) or 250 nM EGFR kinase inhibitor AG1478 (AG). Representative experiment with corresponding micrographs. N: culture medium; P: priming medium; MΦ: supernatant of control macrophages; P-MΦ: supernatant of primed macrophages; scale bar: 200 μm; n = 3; *p < 0.05 by Student’s T-Test. (**B**). OSM promotes motility of MCF7 cells. Cells were incubated for 5 days with or without the indicated concentrations of recombinant OSM. Subsequently, cells were plated into Boyden chambers, using the same dose of OSM in RPMI + 2% FCS in the upper and lower compartments. (**C**). OSM promotes motility of SCC-9 cells. Cells were incubated for 48 hrs with or without 10 ng/ml recombinant OSM, and were subsequently plated into Boyden chambers, using the same dose of OSM in RPMI in the upper and lower compartments. Results are means ± SEM; n = 4; *p < 0.05 by Student’s T-Test. (**D**). HB-EGF and OSM cooperate in promoting chemotaxis of MCF 10A cells. Cells were cultured for 48 hrs with or without the indicated concentrations of recombinant OSM. Subsequently, cells were plated into Boyden chambers, using the same concentration of OSM in RPMI in the upper and lower compartments. Where indicated, 2 ng/ml recombinant HB-EGF was used as a chemoattractant in the lower compartment. Results are means ± SEM; n = 3; *p < 0.05 by Student’s T-Test; micrographs show a representative experiment; scale bar: 200 μm.

In the next set of experiments, we addressed functional aspects of MΦ-derived HB-EGF and OSM on tumor cells. HB-EGF promotes cell migration, invasion, proliferation and survival [15,16]. Clinical and *in vivo* mouse data indicate that HB-EGF drives tumor progression [17-19]. We studied the effect of MΦ-secreted HB-EGF on tumor cell migration in vitro. Supernatants of tumor-primed MΦ increased SCC chemotaxis by 3.8-fold compared to supernatants of control MΦ or priming medium alone. An HB-EGF neutralising antibody or the EGFR inhibitor AG1478 reduced migration to basal levels (Figure 3A).

Though originally reported as an antiproliferative factor [20], OSM failed to inhibit proliferation of several breast cancer cell lines (Additional file 1: Figure S7A). However, OSM induces the undirected motility of MCF7 cells [21]. We incubated MCF7 cells for five days at increasing doses of recombinant OSM. The Boyden chamber assay confirmed a dose-dependent increase in undirected MCF7 cell motility (Figure 3B). Similarly, a 2.6-fold increase in undirected motility was observed in SCC-9 cells (Figure 3C).

10A, benign breast epithelial cells, are barely migratory under most assay conditions. OSM did not induce motility of 10A cells (data not shown). However, OSM-treated 10A cells migrated two to three times faster along an HB-EGF gradient (Figure 3D). For this cell line, HB-EGF is a potent mitogen (Additional file 1: Figure S7B).

Taken together, prolonged treatment of MCF7 and SCC cells with recombinant OSM induces undirected motility in these tumor cells. Priming of MΦ by tumor cells results in HB-EGF secretion at levels sufficient to drive cell migration, and HB-EGF is the relevant chemotactic factor for SCC cells secreted by primed MΦ. OSM and HB-EGF cooperate in promoting chemotaxis of MCF 10A epithelial cells. All three cell lines express receptors for OSM (Additional file 1: Figure S2) and EGFR (Additional file 1: Figure S6A), [22,23].

### TAM express HB-EGF/OSM in invasive breast carcinoma and HB-EGF plasma levels correlate with primary tumor growth and lymph node involvement

**Figure 4:**
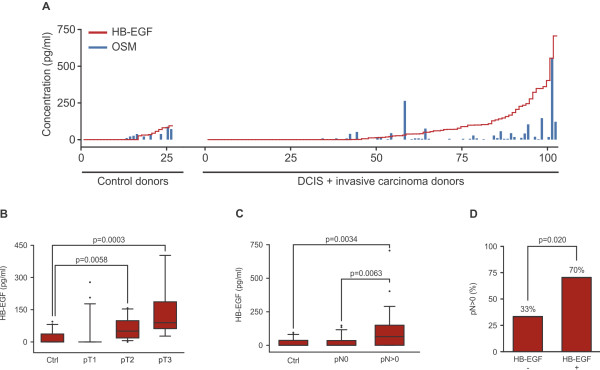
**Systemic protein levels of HB-EGF and OSM are elevated in breast carcinoma patients.** (**A**). HB-EGF and OSM plasma protein levels correlate in human donors. Plasma from 26 control donors and 102 breast carcinoma patients was analysed. Pearson’s correlation coefficient between HB-EGF and OSM levels: 0.722; 95% confidence interval: 0.628 to 0.795; p < 2.2e-16 (alternative hypothesis: true correlation is not equal to 0). (**B**). HB-EGF plasma protein levels correlate with primary tumor size in patients with invasive breast carcinoma. Box plots show HB-EGF plasma levels of 26 control donors, 23 pT1 donors, 13 pT2 donors and 9 pT3 donors. P-values by Mann-Whitney’s U-Test are: Ctrl-pT2: 0.0058; Ctrl-pT3: 0.0003; pT1-pT2: 0.0017; pT1-pT3: 0.0008. (**C**). HB-EGF plasma protein levels correlate with lymph node dissemination of invasive breast carcinoma. Box plots depict HB-EGF levels of control donors (Ctrl; n = 26), donors with nodal negative (pN0; n = 26) and with nodal positive disease (pN > 0; n = 28). (**D**). Elevated HB-EGF plasma levels are indicative of disseminating breast carcinomas. Invasive breast carcinoma patients (n = 54) were grouped into an HB-EGF negative (-; n = 27) and HB-EGF positive (+; n = 27) cohort, using the mean plasma HB-EGF concentration of control donors (21.2 pg/ml; n = 26) as a threshold. 70% (19/27) of HB-EGF positive patients had disseminated tumors, whereas only 33% (9/27) of HB-EGF negative patients were lymph node positive. p = 0.020 by Mann Whitney’s U-Test. Conditional probabilities: P(pN > 0 | HB-EGF+) = 0.704 and P(pN > 0 | HB-EGF-) = 0.333.

According to the TAM/tumor cell interaction model, secreted factors produced by primed TAM might reach the blood stream and be detected in blood samples of cancer patients. Detection of elevated plasma levels of TAM-secreted factors would specify the patient’s tumor status. We determined HB-EGF and OSM plasma levels in mammary carcinoma patients. Plasma samples from 102 female patients with ductal carcinoma *in situ* (DCIS) or invasive breast carcinoma were analysed. 26 healthy female donors served as controls. A highly significant correlation is apparent between plasma HB-EGF and OSM protein levels (Figure 4A and Additional file 1: Table S1; Pearson correlation value: 0.722; 95% confidence interval: 0.628 to 0.795; p = 2.2e^-16^). In healthy donors, HB-EGF and OSM concentrations were constitutively low (mean HB-EGF concentration: 21.2 pg/ml; mean OSM concentration: 13.8 pg/ml). Interestingly, the concerted secretion of both ligands was also detected in two fibroadenoma patients and one patient with Crohn’s disease (Additional file 1: Figure S8).

Given that HB-EGF levels were elevated in a larger proportion of patients’ plasma samples, we reasoned that HB-EGF plasma levels might correlate with growth and/or progression of mammary carcinomas. Mean plasma concentrations of HB-EGF reflect primary tumor size: for small tumors (pT1; 23 patients) we detected 29.6 pg/ml, medium sized tumors (pT2; 13 patients) displayed 62.1 pg/ml and large tumors (pT3; 9 patients) 131.8 pg/ml. In conclusion, HB-EGF plasma levels correlated with primary tumor size (Figure 4B).

In addition, elevated HB-EGF plasma levels also correlate with lymph node dissemination of mammary breast carcinomas (Figure 4C): healthy donors (Ctrl; n = 26) and lymph node negative invasive mammary carcinoma patients (pN0; n = 26) displayed mean HB-EGF plasma concentrations of 21.2 pg/ml and 24.6 pg/ml, respectively, whereas significantly elevated HB-EGF levels were detected in patients with disseminated tumors (97.5 pg/ml; pN > 0; n = 28).

We defined the mean plasma HB-EGF concentration of healthy donors (21.2 pg/ml; Figure 4A) as a threshold that grouped invasive breast carcinoma patients into an HB-EGF negative or HB-EGF positive cohort. In this scoring, 50% (27 of 54) of the patients were determined HB-EGF positive, and tumor cell positive lymph nodes were detected in 52% (28 of 54) of the patients. However, 70% (19 of 27) of HB-EGF positive patients were diagnosed with disseminated tumors, whereas only 33% (9 of 27) of the HB-EGF negative cohort were lymph node positive (Figure 4D). In conclusion, mammary carcinoma patients with disseminated tumors can be identified by elevated HB-EGF plasma levels.

To test a potential participation of monocyte-secreted EREG in the development of mammary tumors, we quantified EREG plasma protein levels in 59 donors with DCIS or invasive mammary carcinomas as well as in 10 healthy donors. In the control donors and in the majority of breast cancer patients, EREG plasma levels remained low (Additional file 1: Figure S9). We only detected elevated EREG levels in 5 of 59 cancer patients, rendering a significant contribution of monocyte-derived EREG to the progression of breast cancer implausible. A supplementary list containing ELISA and clinical patient data is available online (Additional file 2).

**Figure 5:**
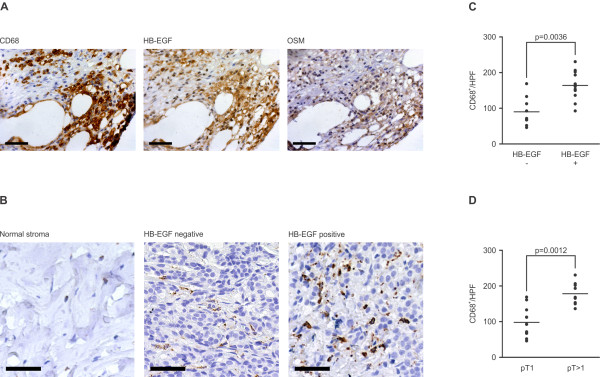
**HB-EGF and OSM are co-expressed by TAM in breast carcinomas.** (**A**) TAM co-express HB-EGF and OSM. Paraffin-embedded tumor specimens of breast carcinoma patients with elevated HB-EGF plasma levels were stained with an antibody detecting the macrophage marker CD68. Consecutive slides were stained with antibodies against HB-EGF and OSM. Representative micrographs. Scale bar: 50 μm. (**B**). Primary tumors of HB-EGF positive breast carcinoma patients are densely infiltrated by TAM. Macrophages were detected by a monoclonal anti-CD68 antibody as in (**A**). Representative micrographs show macrophage infiltrates in tumor samples of HB-EGF positive and negative patients. Healthy breast stromal tissue is largely devoid of macrophage infiltrates. Scale bar: 50 μm. (**C**). Elevated HB-EGF plasma levels correspond to increased TAM density in breast carcinoma patients. TAM in paraffin-embedded primary tumor samples of patients with elevated HB-EGF plasma levels (+; n = 12; mean HB-EGF concentration: 222.1 pg/ml) and of HB-EGF negative patients (-; n = 9) were identified by anti-CD68 staining. TAM numbers were quantified in 6 high power fields per patient (HPF). Scatter plots indicate mean values for each patient (dots) and mean values for both cohorts (horizontal bars). p = 0.0036 by Mann-Whitney’s U-Test. (**D**). TAM density correlates with tumor size in breast carcinoma patients. Tumor-infiltrating macrophages were quantified as in (**C**). Patients were divided into two cohorts according to tumor size (pT1: tumor size ≤ 2 cm; n = 12; pT > 1: tumor size > 2 cm; n = 9). p = 0.0012 by Mann-Whitney’s U-Test.

To test whether TAM express HB-EGF and OSM *in vivo*, we performed immunohistochemical analysis of paraffin-embedded tissue samples of breast cancer patients with elevated HB-EGF plasma levels. Our analysis confirmed that TAM co-expressed HB-EGF and OSM in primary tumors (Figure 5A). Compared to HB-EGF negative patients, we observed an increase in TAM density in tumors of HB-EGF positive patients (Figure 5B). Quantitation of TAM numbers in patients with elevated HB-EGF plasma levels (n = 12) and HB-EGF negative patients (n = 9) revealed a highly significant correlation between HB-EGF plasma levels and TAM infiltration (Figure 5C). Corroborating our previous observation that increased plasma levels of HB-EGF are primarily found in patients with large primary tumors, increased TAM densities also significantly correlate with tumor size (Figure 5D).

Confirming our in vitro interaction model, the presented immunohistochemical data identify TAM as sources of production of HB-EGF and OSM in primary tumors. Elevated HB-EGF and OSM levels in patients’ plasma indicate secretion of both ligands by TAM in mammary carcinomas *in vivo*.

Given the strong correlation between TAM density, HB-EGF plasma levels and tumor size, increased HB-EGF plasma levels identify a substantial part of node positive patients, connecting TAM-secreted HB-EGF with tumor dissemination.

### Tumor *HBEGF* expression is predominantly localised in the stromal compartment

To further substantiate the clinical data, we analysed tumor microarray datasets available at Oncomine™. The breast carcinoma microarray dataset by Ambs and colleagues distinguished between genes expressed by tumor cells versus stromal cells [24]. Our analysis of this dataset revealed that both *HBEGF* and the monocytic marker *CD64* are predominantly expressed in the stromal compartment (Additional file 1: Figure S10A), suggesting that tumor-associated mononuclear cells express this EGFR agonist.

In our experiments, priming of differentiated MΦ was not restricted to mammary carcinoma cell lines. MΦ-derived factors are also functional in other carcinomas. Analysis of the bladder carcinoma microarray dataset published by Radvanyi and colleagues [25] showed *HBEGF* to be overrepresented in infiltrating carcinoma samples (Additional file 1: Figure S10B). These data corroborate our in vitro findings that HB-EGF is involved in the progression of a variety of carcinomas.

## Discussion

Recent scientific advances have established that monocytes and macrophages are not only critically involved in inflammatory processes such as pathogen defence and wound healing, but also play an active role in cancer development. Their functions are modulated by tumor cells, resulting in tolerant, trophic phenotypes indispensable for tumor progression [6]. Among the factors secreted by tumor cells the chemokine CCL2 has been identified as an important priming factor of TAM *in vivo*[26]. Based on a model that tumor cells and mononuclear cells communicate via secreted factors, we have used cell supernatants to study the reciprocal influence of both cell types. Our main focus was on the paracrine release of both EGFR and STAT3 activators by monocytes/macrophages to tumor cells [6]. The presented data indicate that the differentiation process of monocytes to macrophages is of no importance for the activation of these two signaling pathways, even though the precise nature of the secreted ligands changes with differentiation.

Constitutive activation of STAT3 is common in melanoma, breast, head and neck, lung, pancreatic and prostate cancer [27]. Tumor-primed primary PBMC and MΦ secrete oncostatin-M, and only OSM activates STAT3 in our reporter system. Both OSM receptors are exclusively found in epithelial and carcinoma cells. This underscores the unique paracrine role of mononuclear cell-derived OSM acting on tumor cells. This is the first report that shows tumor cell-derived activities to induce OSM secretion by primary human PBMC and MΦ, supporting an interaction model defined by mutually secreted factors. All of the tested tumor cell lines effectively primed human PBMC and/or differentiated MΦ. Priming induced rather complex transcriptional induction profiles of EGFR ligands in mononuclear cells. At protein level, however, priming resulted in co-secretion of EREG and OSM by PBMC, while tumor-primed MΦ co-secreted HB-EGF and OSM in vitro. Transcript analysis alone, however, would not reliably predict the functionally relevant EGFR agonist released by tumor-primed mononuclear cells [28]. In summary, two secretory patterns emerge in PBMC and MΦ in response to tumor cell-secreted factors: a lineage-specific EGFR ligand in combination with OSM.

Functionally, recombinant OSM and HB-EGF strongly cooperate in inducing chemotaxis of benign breast cells in vitro. OSM enhances the intrinsic motility of carcinoma cells, while macrophage-produced HB-EGF is the key chemotactic factor for squamous cell carcinoma cells in our assays. Our in vitro data suggest that TAM-derived OSM and HB-EGF influence migratory processes of cancer cells. In breast cancer patients, HB-EGF and OSM are co-expressed by TAM and plasma protein levels of both ligands correlate strongly. Elevated HB-EGF plasma protein levels are strongly associated with primary tumor growth and lymph node dissemination in invasive breast cancer patients. In fact, the majority of lymph node positive patients display elevated HB-EGF plasma levels. Increased density of infiltrating TAM in patients with elevated HB-EGF plasma levels further supports the hypothesis that TAM-secreted HB-EGF promotes breast carcinoma growth and metastasis *in vivo*. *HBEGF* transcripts primarily detected in the stroma of mammary carcinomas correlate with the myeloid marker *CD64*, further supporting the assumption that TAM account for the expression of *HBEGF* in the tumor microenvironment. Our analysis of the Stransky bladder carcinoma microarray data [25] corroborated these findings inasmuch as *HBEGF* transcript levels increase in infiltrating tumors with respect to superficially growing lesions. In bladder carcinomas, *HBEGF* transcript levels inversely correlate with patients’ survival times [19]. As Massagué and co-workers have shown in a mouse xenograft model, *HBEGF* sustains the specific dissemination of human mammary tumor cells to the brain, whereas dissemination to the lung is accompanied by *EREG* expression [29,30]. Elevated EREG plasma protein levels, however, were detected in a minor fraction of our breast cancer patient data set, suggesting that EREG is not a primary contributor to breast cancer progression mediated by monocytes in humans. Immunohistochemical studies of HB-EGF expression in breast carcinoma tumor cells have found no [31] or an inverse correlation [32] to tumor progression parameters, whereas our study indicates a positive contribution of TAM-derived HB-EGF production in the primary tumor to invasive mammary carcinoma progression. TAM expression of HB-EGF has been reported for cholangiocarcinoma metastases. Additionally, this report linked the priming of mononuclear cells to HB-EGF expression in vitro [33].

Inflammatory processes have been studied in wound healing, and macrophages play a key role in skin repair [34]. In this process, only infiltrating polymorphonuclear neutrophils have yet been described to secrete OSM [35], and HB-EGF is detected in wound exudates [36].

Our work focuses on paracrine factors secreted by mononuclear cells upon priming by tumor cells. This priming process induces secretory patterns that consistently feed back to activate oncogenic EGFR- and STAT3 pathways in tumor cells. We identify HB-EGF and OSM as the key EGFR and STAT3 activators secreted by tumor-primed MΦ, and EREG and OSM as the key activating factors secreted by tumor-primed PBMC. HB-EGF and OSM profoundly influence tumor cell behaviour and cooperatively promote cell motility. Our in vitro results indicate that these proteins effectively mediate macrophage-assisted cancer progression. The identified stereotype secretion patterns are elementary to design strategies to interfere with pro-tumorigenic mononuclear functions by inhibiting HB-EGF, EREG and OSM. In particular, HB-EGF function is apparently connected to tumor growth and dissemination. But first, a clear clinical characterisation of the subset of tumors infiltrated by pro-tumorigenic mononuclear cells is essential. In particular, HB-EGF serves as potential blood-bound marker allowing the minimally invasive identification of tumors supported by TAM. Our work describes pro-tumorigenic marker proteins that identify tumor-supportive mononuclear cells and establishes a basis for therapeutic intervention aimed at neutralising these STAT3 and EGFR activators at the molecular level.

## Conclusions

Soluble tumor-cell-derived factors trigger mononuclear cells to co-secrete lineage-specific EGFR agonists together with a common STAT3 activator. Tumor-primed MΦ release HB-EGF and OSM, while tumor-primed PBMC co-secrete EREG and OSM. TAM co-express HB-EGF and OSM in primary mammary carcinoma samples. In these patients, HB-EGF plasma protein levels strongly correlate with TAM infiltration levels, primary tumor size and lymph node dissemination, indicating that TAM-derived HB-EGF supports carcinoma progression. Thus, HB-EGF plasma protein levels are a potential marker indicating a pro-tumor reaction of TAM in mammary carcinoma.

## Competing interests

The authors declare that they have no competing interests.

## Authors’ contributions

PV designed the study, performed the experiments and drafted the manuscript. PM carried out in vitro experiments. TM planned the patient study and drafted the manuscript. PW and BA planned the patient study and gathered patient samples and clinical data. BH conducted immunohistochemistry experiments. WE planned the patient study, supervised the data and drafted the manuscript. PK performed in vitro experiments and designed the study. AU designed the study, supervised the data and drafted the manuscript. All authors read and approved the final manuscript.

## Pre-publication history

The pre-publication history for this paper can be accessed here:

http://www.biomedcentral.com/1471-2407/13/197/prepub

## Supplementary Material

Additional file 1 Supporting information. Click here for file

Additional file 2 Data of patients and control donors. Click here for file
