# Supplementary material for: Monocytes/macrophages support mammary tumor invasivity by co-secreting lineage-specific EGFR ligands and a STAT3 activator
Source: BMC Cancer. 2013 Apr 18;13:197. doi: 10.1186/1471-2407-13-197 (PMC3648435; doi:10.1186/1471-2407-13-197)
Supplement: Additional file 1 — Supporting information. [file 1471-2407-13-197-S1.doc]

**SUPPORTING INFORMATION**

**Inventory**

1. **SUPPORTING DATA**

**Figure S1**

**Figure S2**

**Figure S3**

**Figure S4**

**Figure S5**

**Figure S6**

**Figure S7**

**Figure S8**

**Figure S9**

**Figure S10**

**Table S1**

1. **SUPPORTING EXPERIMENTAL PROCEDURES**

Monocyte isolation by positive selection

Monocyte/macrophage priming

Proliferation assays

Stimulation of cells and immunoblotting

ELISA

Immunohistochemistry

Gene expression analysis

Flow cytometry

Patient sample collection

1. **SUPPORTING REFERENCES**

Two supplementary references

1. **SUPPORTING DATA**

**
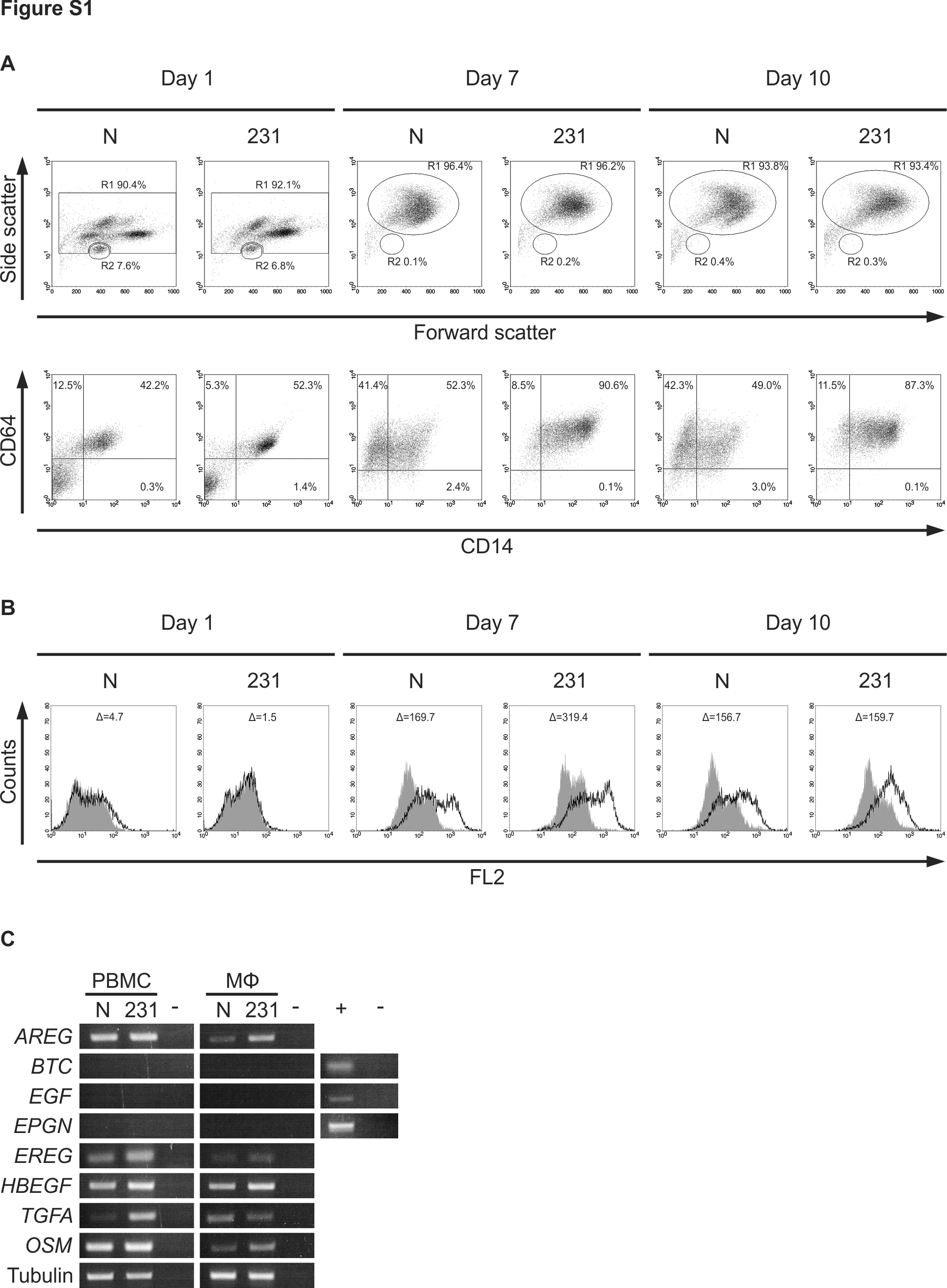
**

**Figure S1. PBMC differentiate *in vitro* to MΦ, and both cell types express EGFR ligands and OSM.**

1. PBMC and differentiated macrophages express CD64 and CD14. Freshly isolated PBMC were treated with normal culture medium (N) or MDA-MB-231-conditioned medium (231) for 24 hrs. Alternatively, cells were allowed to differentiate in normal culture medium for 4 days, after which they received culture medium or MDA-MB-231-conditioned medium for 6 days. At the indicated timepoints, CD14 FITC/CD64 PE double-stained cells were analysed in a flow cytometer. R1: monocytic region; R2: lymphocytic region.
2. Scavenger receptor AI expression accompanies the *in* *vitro* differentiation of PBMC to MΦ. PBMC differentiated *in vitro* over a 10-day period as described in (A). At the indicated timepoints, SR-AI expression was assayed in a flow cytometer. Δ: difference between the median fluorescence intensities of stained cells and cells treated with control Ig.
3. PBMC and primary human MΦ express EGF family ligands and OSM. Freshly isolated PBMC and *in* *vitro* differentiated MΦ were treated with normal culture medium (N) or MDA-MB-231-conditioned medium (231) for 24 hrs. PCR was performed on reverse-transcribed mRNA samples. -: water control; +: placenta cDNA positive control.


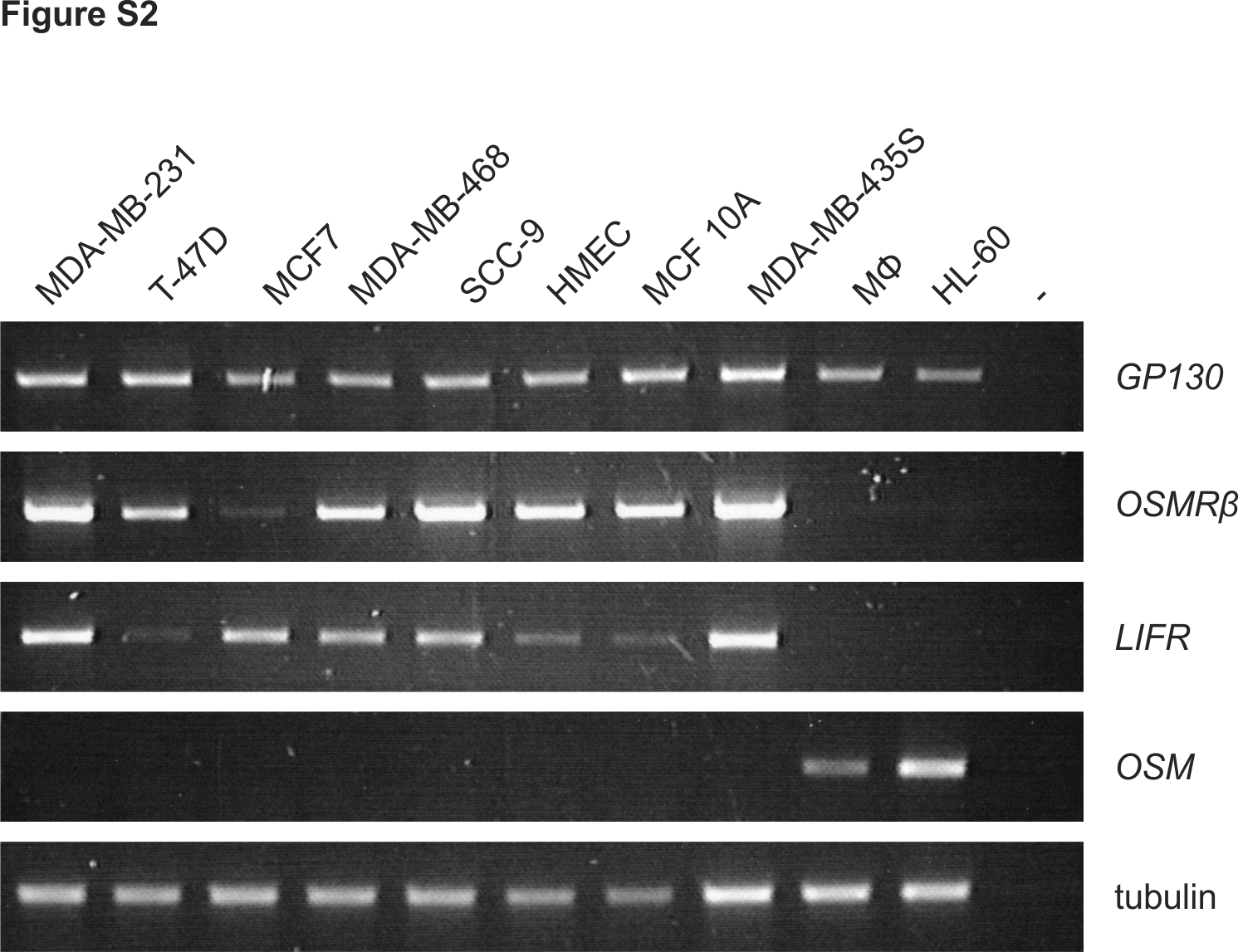


**Figure S2. Myeloid cells and epithelial cells differentially express OSM and OSM receptors.**

*OSM* is expressed in primary human macrophages (MΦ) and HL-60 myelocytes, OSM receptors are widely expressed in tumor cell lines and nontransformed mammary epithelial cells (RT-PCR). -: water control.


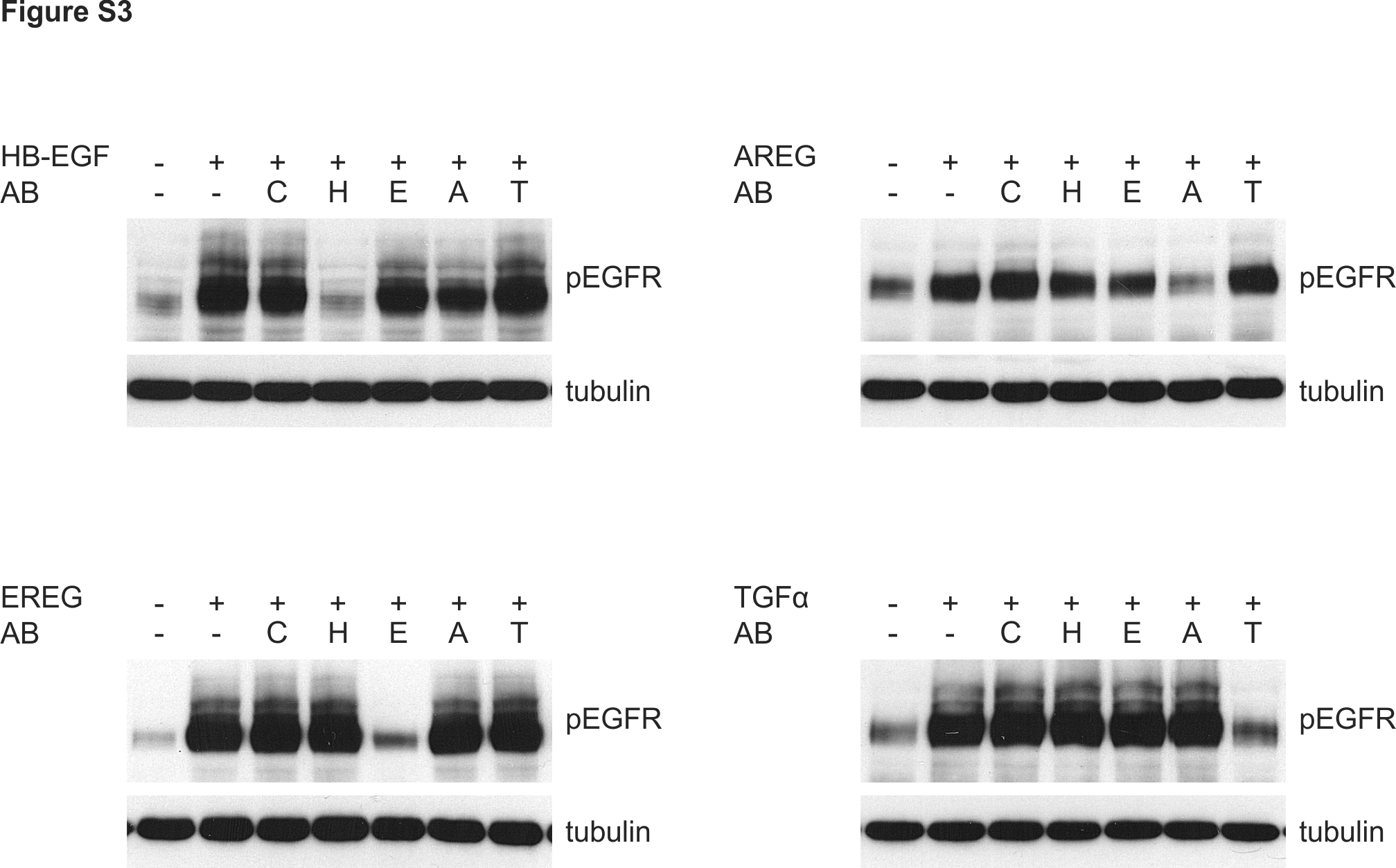


**Figure S3. Neutralizing antibodies against HB-EGF, EREG, AREG and TGFα show no significant cross-reactivity.**

SCC-9 cells were stimulated with culture medium supplemented with recombinant human HB-EGF (10ng/ml), EREG (2ng/ml), AREG (10ng/ml) or TGFα (2ng/ml). Prior to stimulation, media were incubated for an hour with neutralizing antibodies directed against HB-EGF (H), EREG (E), AREG (A), TGFα (T) or with control Ig (C).

**
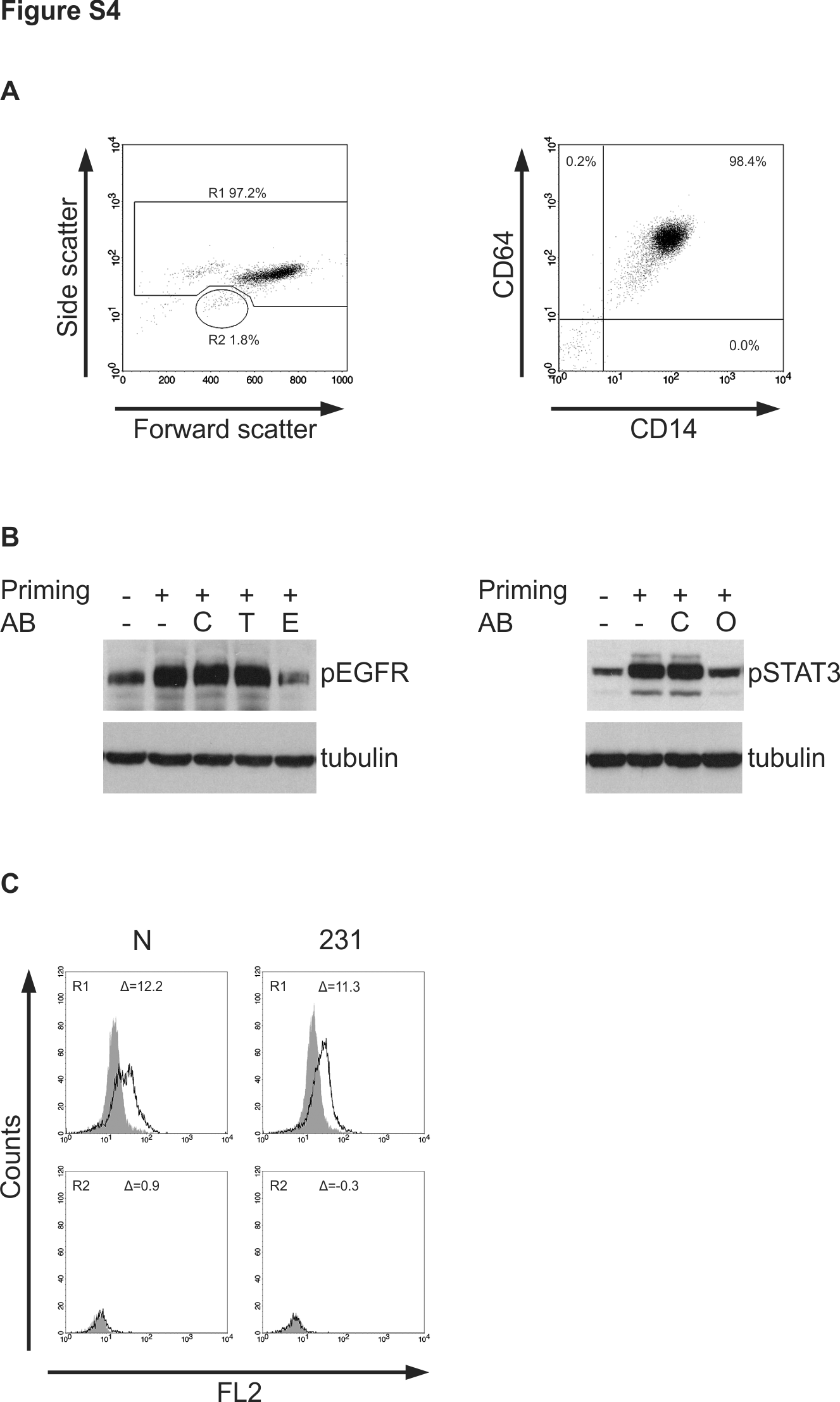
**

**Figure S4. Primary human PBMC secrete EREG and OSM upon priming by MDA-MB-231 carcinoma cells.**

1. PBMC isolated by CD14 positive selection express CD64 and CD14. Freshly isolated PBMC double-stained with CD14 FITC/CD64 PE antibodies were analysed in a flow cytometer. Left panel: forward and side scatter properties. R1: monocytic region; R2: lymphocytic region. Right panel: CD14/CD64 staining (gate = R1).
2. CD14^+^/CD64^+^ PBMC primed by tumor cells secrete EREG and OSM. PBMC isolated by CD14 positive selection received culture medium or MDA-MB-231-conditioned medium for 24 hrs. PBMC supernatants were preincubated with control Ig or blocking antibodies, and were then used to stimulate reporter cells in which pEGFR, pSTAT3 and tubulin levels were visualised. Representative Western blot experiment. C: control Ig; T: TGFα blocking antibody; E: EREG blocking antibody; O: OSM blocking antibody.
3. Primary human PBMC express pro-EREG. PBMC were isolated via cell adherence and received culture medium (N) or MDA-MB-231-conditioned medium (231) for 24 hrs. EREG expression levels were visualised via flow cytometry of cells stained with an epiregulin PE antibody. R1: monocytic region; R2: lymphocytic region.

**
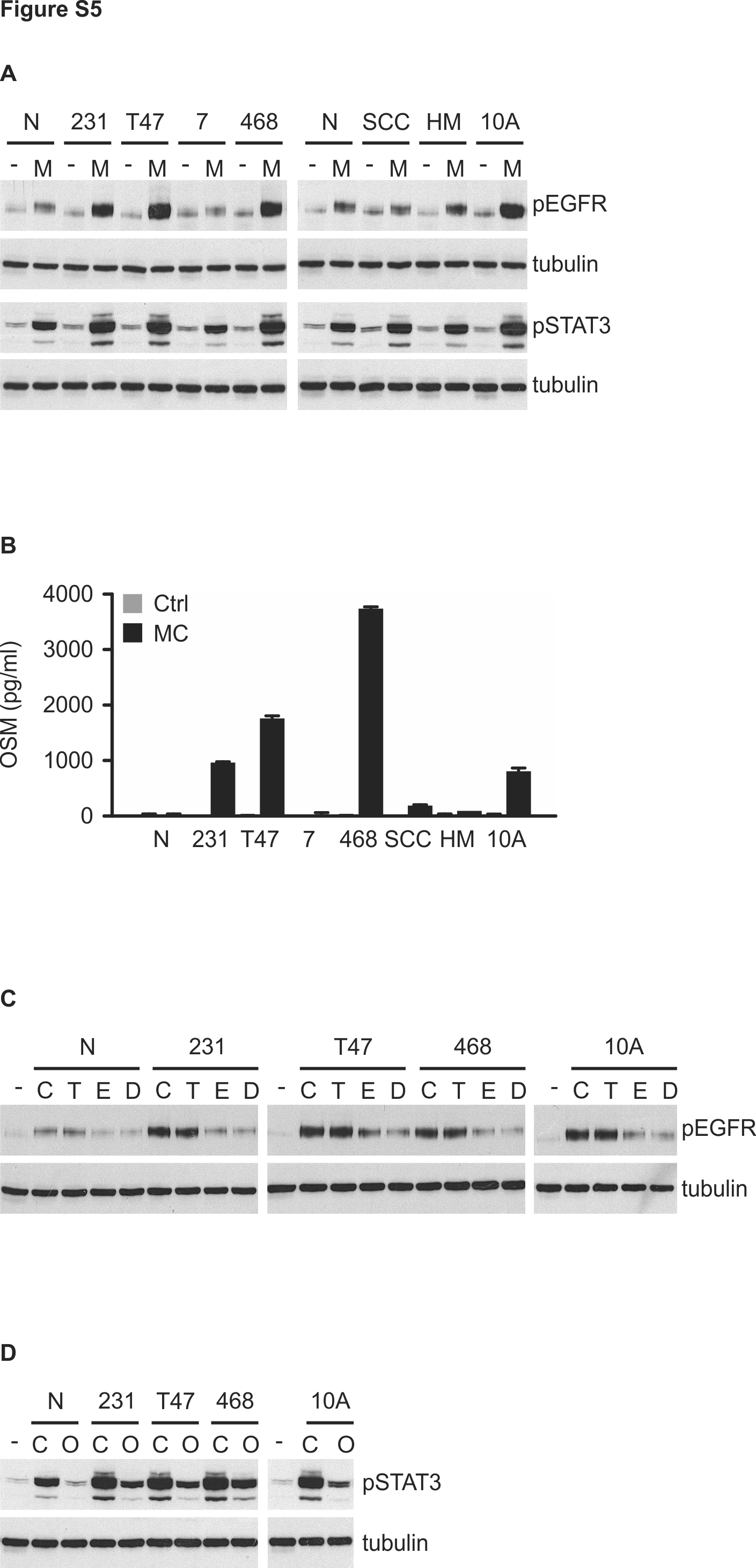
**

**Figure S5. Tumor-primed PBMC secrete EREG and OSM.**

1. Supernatants of primed PBMC contain EGFR and STAT3 activators. Reporter cells were stimulated with either normal culture medium (N) or with supernatants from a panel of cell lines (231, T47, 7, 468, SCC, HM, 10A). Prior to stimulation of reporter cells, all media and supernatants were either control-incubated for 24 hrs (-) or incubated with PBMC for the same duration (M). Basal phosphorylation of EGFR and STAT3 is depicted in columns marked by “-“. Signals in “M” represent phosphorylation of EGFR and STAT3 induced by PBMC-secreted factors upon priming with cell line-conditioned media or normal culture medium. Tubulin served as loading control.
2. Primed PBMC secrete OSM (ELISA). Ctrl: priming media that were not incubated with PBMC; MC: PBMC supernatants.
3. EREG is the relevant EGFR agonist secreted by primed PBMC. Reporter cells were stimulated with normal culture medium that was control-incubated for 24 hrs (-) or with supernatants of PBMC primed by normal culture medium (N) or by the indicated cell line-derived supernatants (231, T47, 468, 10A) for 24 hrs. pEGFR levels were visualised, tubulin served as loading control. Where indicated, PBMC supernatants were preincubated with control Ig (C) or the following blocking antibodies: αTGFα (T), αEREG (E), double treatment (D).
4. OSM is the relevant STAT3 activator secreted by primed PBMC. Reporter cells were stimulated with supernatants of PBMC as in (C). pSTAT3 levels were visualised, tubulin served as loading control. Where indicated, PBMC supernatants were preincubated with control Ig (C) or an OSM blocking antibody (O).

**
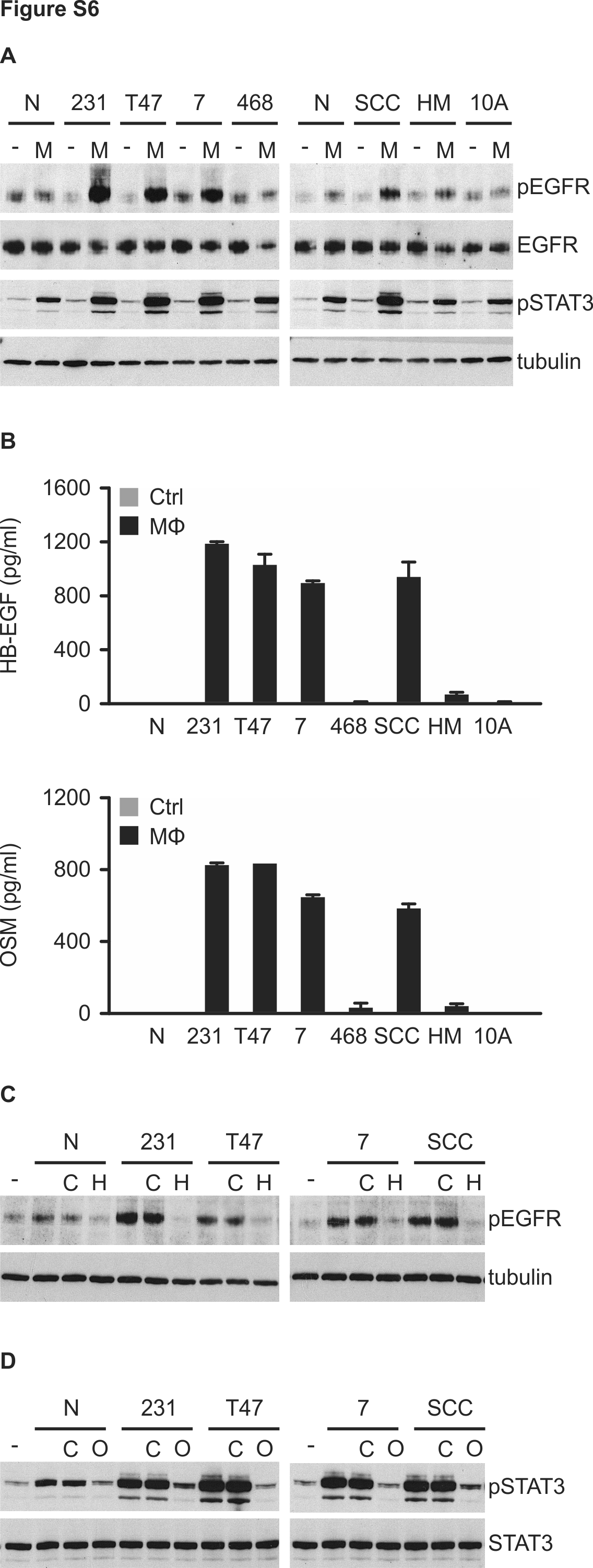
**

**Figure S6. Tumor-primed macrophages secrete HB-EGF and OSM.**

1. Supernatants of primed MΦ contain EGFR and STAT3 activators. Reporter cells were stimulated with either normal culture medium (N) or with supernatants from a panel of cell lines (231, T47, 7, 468, SCC, HM, 10A). Prior to stimulation of reporter cells, all media and supernatants were either control-incubated for 24 hrs (-) or incubated with macrophages for the same duration (M). Basal phosphorylation of EGFR and STAT3 is depicted in columns marked by “-“. Signals in “M” represent phosphorylation of EGFR and STAT3 induced by macrophage-secreted factors upon priming with cell line-conditioned media or normal culture medium. EGFR and tubulin served as loading controls.
2. Primed MΦ secrete HB-EGF and OSM (ELISA). Ctrl: priming media that were not incubated with macrophages; MΦ: macrophage supernatants.
3. HB-EGF is the relevant EGFR agonist secreted by primed MΦ. Reporter cells were stimulated with normal culture medium that was control-incubated for 24 hrs (-) or with supernatants of MΦ primed by normal culture medium (N) or by the indicated cell line-derived supernatants (231, T47, 7, SCC) for 24 hrs. pEGFR levels were visualised, tubulin served as loading control. Where indicated, MΦ supernatants were preincubated with control Ig (C) or with an HB-EGF blocking antibody (H).
4. OSM is the relevant STAT3 activator secreted by primed MΦ. Reporter cells were stimulated with supernatants of MΦ as in (C). pSTAT3 levels were visualised, STAT3 served as loading control. Where indicated, MΦ supernatants were preincubated with control Ig (C) or an OSM blocking antibody (O).

**
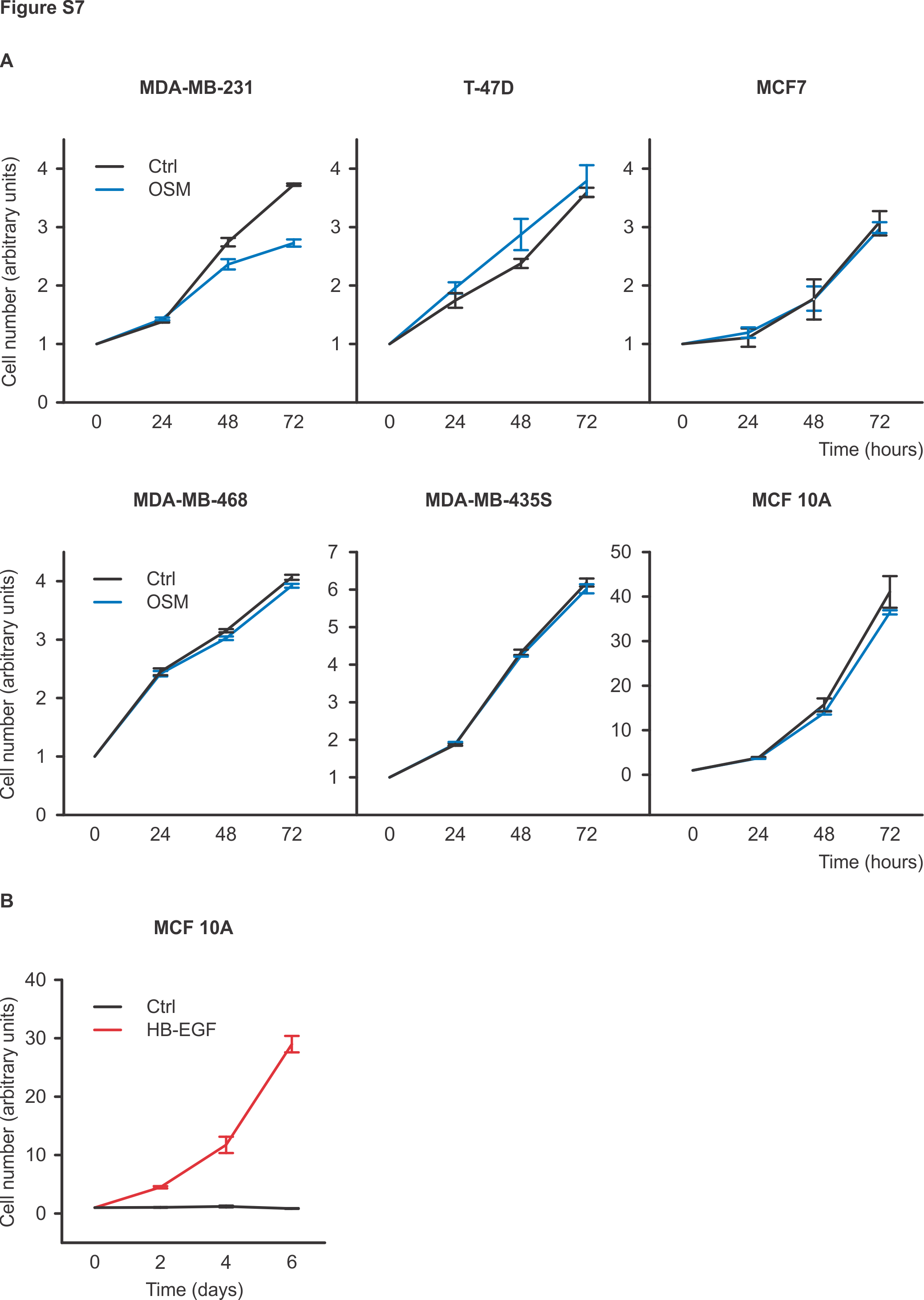
**

**Figure S7. HB-EGF, but not OSM, has a major impact on breast epithelial cell proliferation.**

1. Oncostatin-M lacks potent antiproliferative activity in breast tumor cells. Cells were cultured in their respective culture media, with or without 20 ng/ml recombinant OSM. At the indicated time points, cells were detached and counted.
2. HB-EGF induces proliferation of MCF 10A cells. Cells were cultured in serum-free medium, with or without 40 ng/ml recombinant HB-EGF. At the indicated time points, cells were detached and counted.


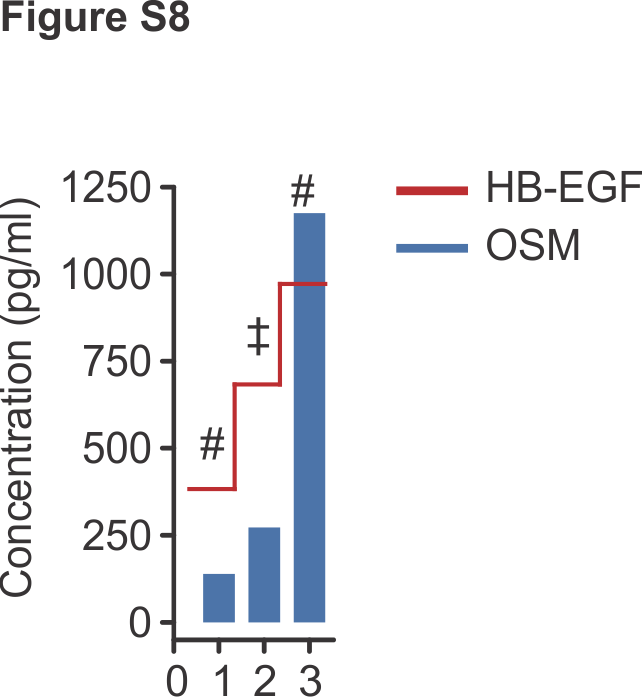


**Figure S8. HB-EGF and OSM are co-secreted in patients with fibroadenoma and Crohn’s disease.**

HB-EGF and OSM plasma levels were determined in one patient with Crohn’s disease (‡) and two patients with fibroadenoma (#).

**
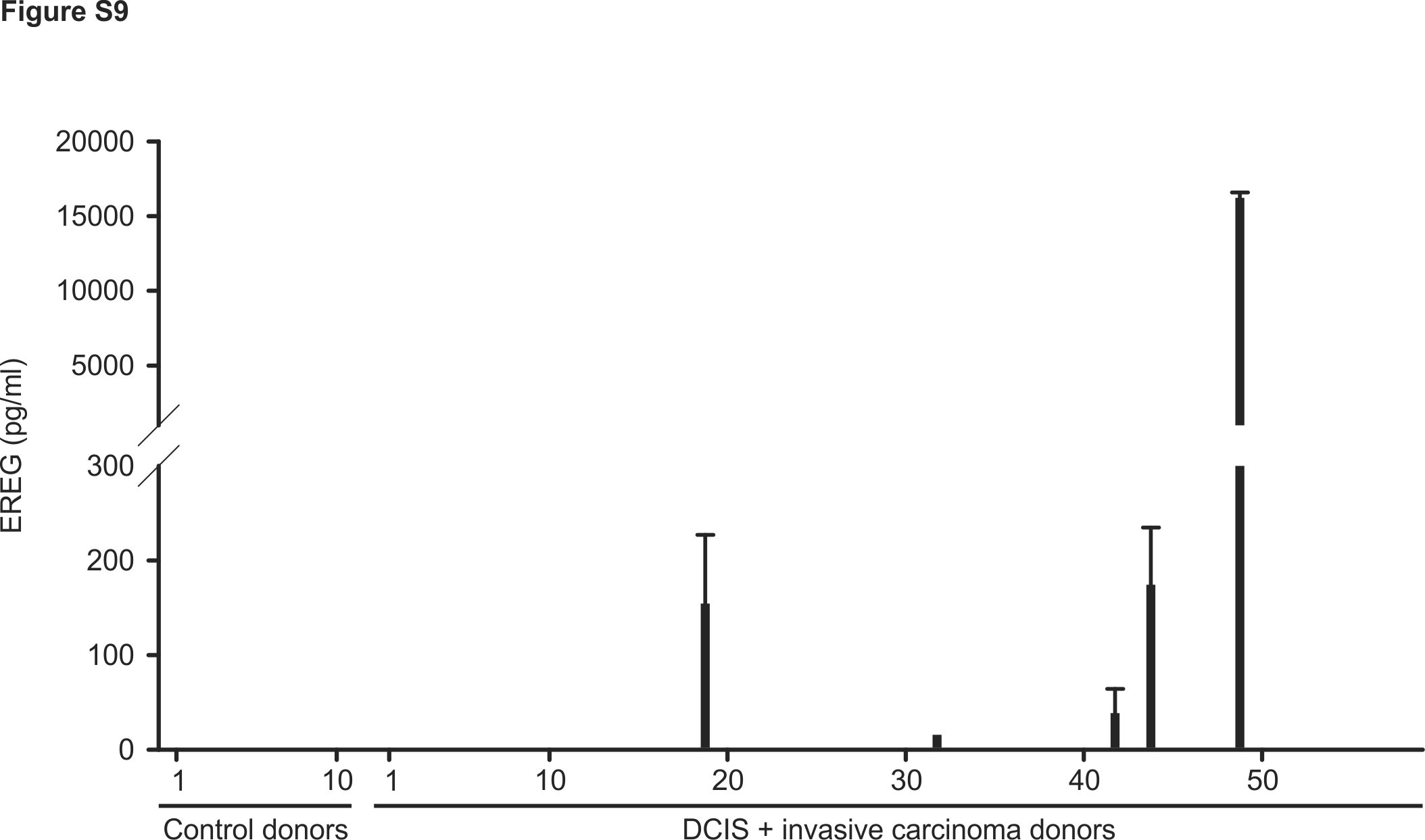
**

**Figure S9. EREG plasma protein levels are elevated among breast cancer patients.**

Plasma from 10 control donors and 59 breast carcinoma patients was analysed (ELISA).

**
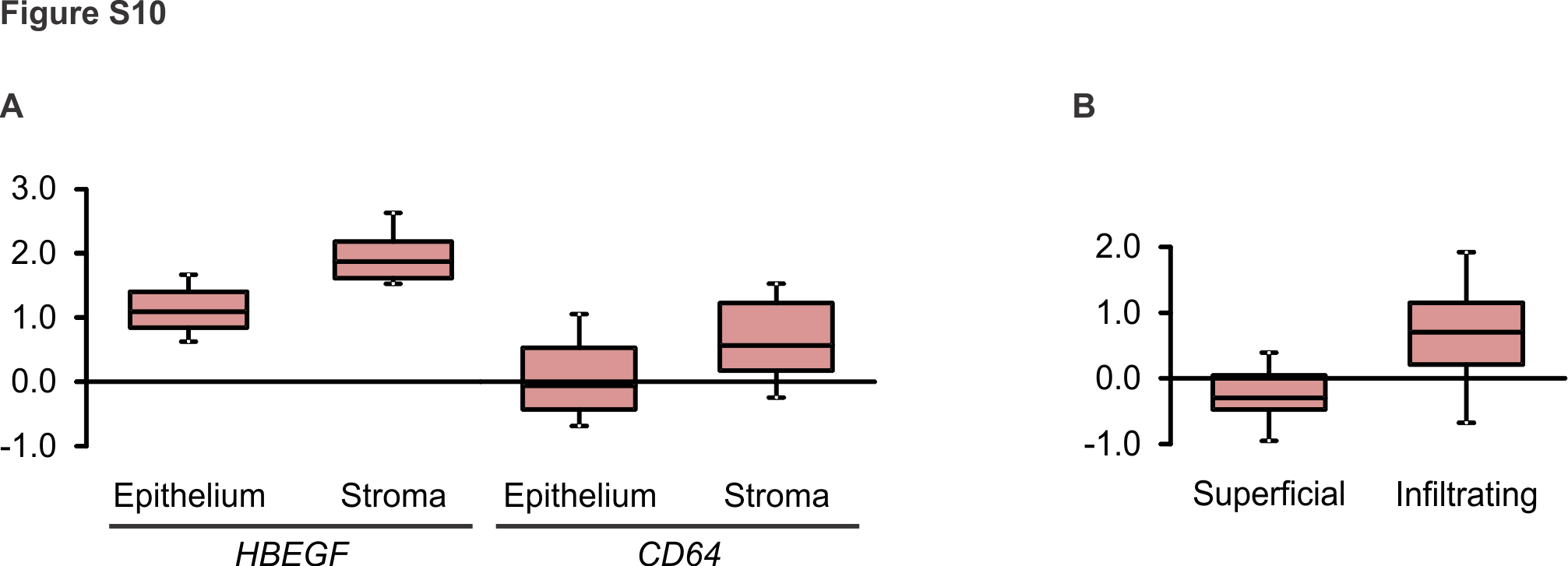
**

**Figure S10. *HBEGF* is expressed in the stromal compartment of breast carcinomas and in infiltrating bladder carcinoma.**

1. *HBEGF* and the myeloid marker *CD64* are predominantly expressed in the stromal compartment of breast carcinomas. Boxplots show Oncomine^TM^ data analysis of the Boersma breast microarray dataset. Tumor samples were subjected to laser capture microdissection and differential microarray analysis was performed on epithelium (n=48) versus stroma (n=47).
2. *HBEGF* is predominantly expressed in infiltrating bladder carcinoma. Oncomine^TM^ data analysis of the Stransky bladder microarray dataset shows the expression of *HBEGF* in infiltrating bladder carcinoma (n=32) versus superficial carcinomas (n=25).

**Table S1. HB-EGF and OSM protein plasma levels correlate in patients.**

| **Method** | **Correlation value** | **95% confidence interval** | **p-Value** |
| --- | --- | --- | --- |
| Pearson’s product-moment correlation | 0.722 | 0.628 – 0.795 | <2.2e-16 |
| Kendall’s rank correlation tau | 0.437 | N.D. | 4.4e-10 |

1. **SUPPORTING EXPERIMENTAL PROCEDURES**

**Monocyte isolation by positive selection**

CD14^+^ monocytes were isolated from fresh blood samples using magnetic nanobeads coated with a CD14 specific antibody, according to the manufacturer’s instructions (Miltenyi Biotech, Bergisch Gladbach, Germany).

**Monocyte/macrophage priming**

To obtain media for PBMC/MΦ priming experiments, cells were plated into 15 cm culture plates, using their respective culture media. After 24 hrs, 60% confluent cells were washed 3 times with PBS and incubated in 20 ml monocyte culture medium for 24 hrs. Conditioned media were collected, filtered through a 0.22 µm filter, shockfrozen and stored at -70 °C. For priming of PBMC/MΦ, a 1:1 mixture of conditioned medium and fresh monocyte culture medium was used. Priming of PBMC was done for 24 hrs immediately after isolation. For priming of differentiated macrophages, cells were incubated in control medium or priming medium for 24 hrs from day 10 to 11.

**Proliferation assays**

1.5 x 10^5^ cells were plated per well in 6 well culture plates (MDA-MB-231, MDA-MB-435S, MDA-MB-468, T-47D, MCF7) or 2.5 x 10^4^ cells were plated per well in 12 well culture plates (MCF 10A). At the indicated timepoints, cells were detached using Trypsin/EDTA (0.05%/0.02% in PBS; PAA, Pasching, Austria) and counted with a Z1 Coulter^TM^ Particle Counter (Beckman Coulter, Krefeld, Germany).

**Stimulation of cells and immunoblotting**

For stimulation experiments, 1 x 10^5^ MDA-MB-231 or 8 x 10^4^ SCC-9 cells were plated per well in 48 well plates and 24 well plates, respectively (Corning). Following overnight incubation, MDA-MB-231 cells were stimulated for 10 minutes with 100 µl of monocyte/macrophage-conditioned medium or control medium. SCC-9 cells were stimulated for 3 minutes with 200 µl of medium. Where indicated, media have been preincubated for 1 hr at 37 °C with neutralising antibodies, control sera, DMSO or the EGFR inhibitor AG1478 (Alexis Biochemicals, Plymouth Meeting, PA). AREG, EREG and HB-EGF were neutralised with 10 µg/ml affinity-purified goat polyclonal antibodies (catalog #. AF262, [AF1195](http://www.rndsystems.com/pdf/af1195.pdf), AF-259-NA, R&D Systems, Minneapolis, MN). TGFα was neutralised with 10 µg/ml of a goat polyclonal antibody (catalog # AB-239-NA, R&D Systems). OSM was neutralised with 0.3 µg/ml of an affinity-purified goat polyclonal antibody (catalog # AF-295-NA, R&D Systems). Normal goat Ig for control incubations was from R&D Systems (catalog # AB-108-C). Cells were lysed with a buffer containing 50 mM HEPES (pH 7.5), 150 mM NaCl, 1% Triton X-100, 1 mM EDTA, 10% glycerol, 10 mM Na_4_P_2_O_7_, 2 mM Na_3_VO_4_, 10 mM NaF, 1 mM PMSF and 10 µg/ml aprotinin. Lysates were precleared by centrifugation at 17,000 g, 4 °C for 10 minutes. After SDS-polyacrilamide gel electrophoresis, proteins were transferred onto nitrocellulose membranes. Western blotting was performed according to standard procedures. Polyclonal antibodies against phospho-EGFR (catalog # 4407, Tyr 1173), phospho-STAT3 (catalog # 9131, Tyr 705) and STAT3 (catalog # 9132) were from Cell Signaling Technology (Danvers, MA). The EGFR was detected with a polyclonal antibody (catalog # 04-338) from Upstate Biotechnology (Lake Placid, NY). α-Tubulin levels were visualised with a monoclonal antibody from Sigma (catalog # T9026). Unless stated otherwise, the data shown are representative of at least three independent experiments. Densitometry was performed using the Advanced Image Data Analysis software version 4.03.031 (Raytest, Straubenhardt, Germany).

**ELISA**

Nunc MaxiSorp^TM^ 96 well plates (Thermo Fisher Scientific, Waltham, MA) were coated with capture antibodies at room temperature overnight. Plates were washed 4 times with washing buffer containing 0.05% Tween_20_ in PBS, pH 7.4. Next, plates were incubated for 2 hrs at room temperature with 150 µl/well blocking buffer containing 5% sucrose, 1% BSA in PBS. Following 4 washing steps, 100 µl of 1:10 diluted samples or concentration standard were added per well. Concentration standards were prepared in PBS using 10% control plasma or culture medium. After 2 hrs of incubation at room temperature, plates were washed 6 times and 100 µl of biotinylated detection antibodies were applied for 2 hrs. Samples were washed 6 times, incubated with 50 ng/ml peroxidase-streptavidin (Jackson ImmunoResearch, West Grove, PA) for 15 minutes, followed by 6 further washing steps. The colorimetric reaction was performed in 100 µl of TMB substrate (Merck, Darmstadt, Germany) and stopped by addition of 50 µl 1 M H_2_SO_4_. The optical densities at 405 and 570 nm were measured with a spectrophotometer. HB-EGF capture was performed using a monoclonal antibody (a gift from U3 Pharma, Martinsried, Germany) or an affinity-purified goat polyclonal antibody (catalog # AF-259-NA, R&D Systems), with very similar results. OSM and AREG were captured using monoclonal antibodies from R&D Systems (catalog # MAB295, MAB262). A goat polyclonal anti-TGFα capture antibody and all biotinylated detection antibodies were from R&D Systems (catalog # AF-239-NA, BAF259, BAF295, BAF262, BAF239). Antibody and streptavidin dilutions were prepared using 1% BSA in PBS. Human EREG was quantified with a sandwich ELISA kit from USCNK following manufacturer instructions (catalog # E91945Hu, Wuhan, China).

**Immunohistochemistry**

Two micron sections were cut from paraffin-embedded tissue samples. After deparaffinization and hydration endogenous peroxidases were quenched using H_2_O_2_ and antigens were retrieved by boiling in citrate buffer. Following primary antibodies were applied: anti-HB-EGF affinity-purified goat polyclonal, 1:100, 24 hrs (catalog # AF-259-NA, R&D Systems); anti-OSM mouse monoclonal, 1:100, 24 hrs (catalog # MAB295, R&D Systems); anti-CD68 mouse monoclonal (clone PG-M1), 1:100, 1 hr (Dako, Hamburg, Germany). HB-EGF was visualised using the Vectastain ABC kit (Vector Laboratories, Burlingame, CA), and OSM and CD68 were visualised using the Polymer HRP One-Step kit (Zytomed, Berlin, Germany). Prior to mounting samples were counterstained with hematoxylin and dehydrated. TAM numbers were quantified in 6 high power fields per anti-CD68 stained sample.

**Gene expression analysis**

Total RNA was isolated using the Quiagen RNeasy Mini Kit (Quiagen, Hilden, Germany) and reverse-transcribed with AMV reverse transcriptase (Roche, Mannheim, Germany). Semiquantitative PCR was performed using the ReadyMix^TM^ redtaq^TM^ PCR reaction mix (Sigma). PCR products were subjected to electrophoresis in 2% agarose gels and were visualised by ethidium bromide staining. Quantitative PCR was performed on a StepOnePlus^TM^ instrument using the Fast SYBR® Green Master Mix (Applied Biosystems, Foster City, CA). Data analysis and calculation of 95% confidence intervals was performed using the StepOne^TM^ Software version 2.0. Following primer sequences were used: *OSM* semiquantitative PCR: TGGGGGTACTGCTCACACAG and CCTCAGGGTCTCCTCACTGG; *OSM* qPCR: CAAGGCCTG- GATGTTCCTAA and CCAGACCTCTCCAAATCCTG; *EGF*: GCCAAGCAGTCTGTGATTGA and CTGAT- GGCATAGCCCAATCT; *TGFA*: TGTGTCTGCCATTCTGGGTA and GACCTGGCAGCAGTGTATCA; *EREG*: GGGGAGGAGGATGGAGATG and TGAGGACTGCCTGTAGAAGATG; *EPGN*: TGACAGCACT-GACCGAAGAG and AATCCAACACCAATCCCAAT; *BTC*: GAAACTAATGGCCTCCTCTGTG and TCCG-CTTTGATTGTGTGG; *OSMRβ*: GGCA-CAGAGGGTGGATTCTC and GTGTTCATCCGGAGTCGTGA; *LIFR*: TGACTGGAGCCCTGTGAAGA and GCGGATTGTGAGCATTCAAA; *GP130*: TGCCTCCTTTTG-AAGCCAAT and CCTTTGGAAGGTGGAGCTTG; Cyclophilin A: GCCG-CGTCTCCTTTGAGCT and CACCACATGCTTGCCATCC. Primers for Tubulin alpha1 were described previously , as were primers for *HBEGF* and *AREG* .

**Flow cytometry**

Following detachment, cells were fixed with 2% paraformaldehyde. All further steps were carried out on ice. Fc receptors were blocked by a 10 minute incubation step with mouse IgG_2B_ or donkey IgG. Cells were incubated with primary antibodies for 45 minutes, washed twice with PBS + 2% FCS and incubated with fluorescence-labelled secondary antibodies for 45 minutes. After 2 washing steps, cells were analysed in a FACSCalibur flow cytometer (BD Biosciences). Data analysis was done using CellQuest Pro software (BD Biosciences). CD14/CD64 double staining was performed with prelabelled BD Oncomark CD14 FITC/CD64 PE mouse monoclonal antibodies (catalog # 341124, BD Biosciences). The Simultest^TM^ Control prelabelled antibody mixture was used (catalog # 340394, BD Biosciences). Epiregulin was stained with a PE-labelled mouse monoclonal antibody and PE-labelled mouse isotype control (catalog # IC14251P, IC0041P, R&D Systems). Scavenger Receptor AI staining was done with a mouse monoclonal primary antibody (catalog # MAB2708, R&D Systems) and a PE-labelled donkey anti-mouse IgG secondary antibody F(ab)2 fragment (catalog # 715-116-150, Jackson ImmunoResearch). Mouse isotype control antibody was catalog # MAB0041 from R&D Systems, donkey IgG was catalog # 017-000-002 from Jackson ImmunoResearch.

**Patient sample collection**

Plasma and tissue samples of human donors were obtained in accordance with the guidelines of the local ethics committee and after obtaining informed consent. Patient samples were collected at the Red Cross Hospital, Munich, Germany. Samples from control donors were collected at the Red Cross Hospital and at the Max Planck Institute of Biochemistry (Martinsried, Germany). After collection of peripheral blood into heparinized tubes, cells were pelleted at 2,500 g, 4 °C for 15 minutes. Plasma was cleared at 17,000 g, 4 °C for 15 minutes, aliquoted, shockfrozen and stored at -70 °C. Prior to ELISA, samples were heat-inactivated and cleared at 17,000 g, 4 °C for 15 minutes.

1. **SUPPORTING REFERENCES**

1. Hutterer M, Knyazev P, Abate A, Reschke M, Maier H, Stefanova N, Knyazeva T, Barbieri V, Reindl M, Muigg A *et al*: **Axl and growth arrest-specific gene 6 are frequently overexpressed in human gliomas and predict poor prognosis in patients with glioblastoma multiforme**. *Clin Cancer Res* 2008, **14**(1):130-138.

2. Gschwind A, Hart S, Fischer OM, Ullrich A: **TACE cleavage of proamphiregulin regulates GPCR-induced proliferation and motility of cancer cells**. *EMBO J* 2003, **22**(10):2411-2421.
